# 1174. Phase 3 Study to Evaluate the Safety, Tolerability, and Immunogenicity of Catch-up Vaccination Regimens of V114 in Healthy Infants, Children, and Adolescents (PNEU–PLAN)

**DOI:** 10.1093/ofid/ofab466.1367

**Published:** 2021-12-04

**Authors:** Natalie Banniettis, Jacek Wysocki, Leszek Szenborn, Wanatpreeya Phongsamart, Punnee Pitisuttithum, Mika Rämet, Ron Dagan, Lori Good, Melanie Papa, Yaru Shi, Luwy Musey, Kara Bickham, Gretchen Tamms, Richard McFetridge, Robert Lupinacci

**Affiliations:** 1 Merck & Co., Inc., Kenilworth, New Jersey; 2 Poznań University of Medical Sciences, Poznán, Wielkopolskie, Poland; 3 Wroclaw Medical University, Wroclaw, Dolnoslaskie, Poland; 4 Mahidol University, Bangkok, Krung Thep, Thailand; 5 Tampere University, Tampere, Pirkanmaa, Finland; 6 Ben-Gurion University of the Negev, Beer Sheva, HaDarom, Israel; 7 Merck & Co., Inc, North Wales, Pennsylvania

## Abstract

**Background:**

Despite widespread use of pneumococcal conjugate vaccines (PCVs) in children, morbidity and mortality caused by pneumococcal disease (PD) remain high, in part due to the emergence of disease caused by non-vaccine serotypes (STs). In addition, many children do not receive the recommended number of PCVs on schedule and, therefore, are at risk for PD. V114 is an investigational 15-valent PCV that contains two epidemiologically important STs, 22F and 33F, in addition to the 13 STs present in the licensed 13-valent PCV (PCV13; Prevnar 13^™^). This Phase 3 descriptive study evaluated the safety and immunogenicity of V114 and PCV13 when given as catch-up vaccination in children who are pneumococcal vaccine-naïve or previously immunized with lower valency PCVs.

**Methods:**

Solicited adverse events (AEs) were collected for 14 days after each vaccination. Serious adverse events (SAEs) were collected throughout study participation. Immunogenicity was evaluated by anti-pneumococcal polysaccharide ST-specific immunoglobulin G (IgG) geometric mean concentrations (GMCs) at 30 days post-last vaccination.

**Results:**

606 healthy children, aged 7 months through 17 years, were randomized (double-blind) to receive V114 (n=303) or PCV13 (n=303) via age-appropriate catch-up vaccination schedules (Table 1). V114 had an acceptable safety profile and was well tolerated. Similar proportions of children aged 7–11 months and 2–17 years reported AEs in the V114 and PCV13 groups. A larger proportion of children aged 12–23 months reported AEs in the V114 group (79%) than the PCV13 group (59%). The proportion of children who reported SAEs was comparable among vaccination groups (V114 and PCV13, respectively, 7–11 months: 10.9%, 7.8%; 12–23 months: 6.5%, 6.3%; 2–17 years: 2.3%, 2.3%). No SAEs were reported to be vaccine-related, and no deaths occurred. At 30 days after the last PCV dose, ST-specific IgG GMCs were comparable for the 13 shared STs and were higher in the V114 group for 22F and 33F.

Table 1. Catch-up vaccination schedules in V114-024

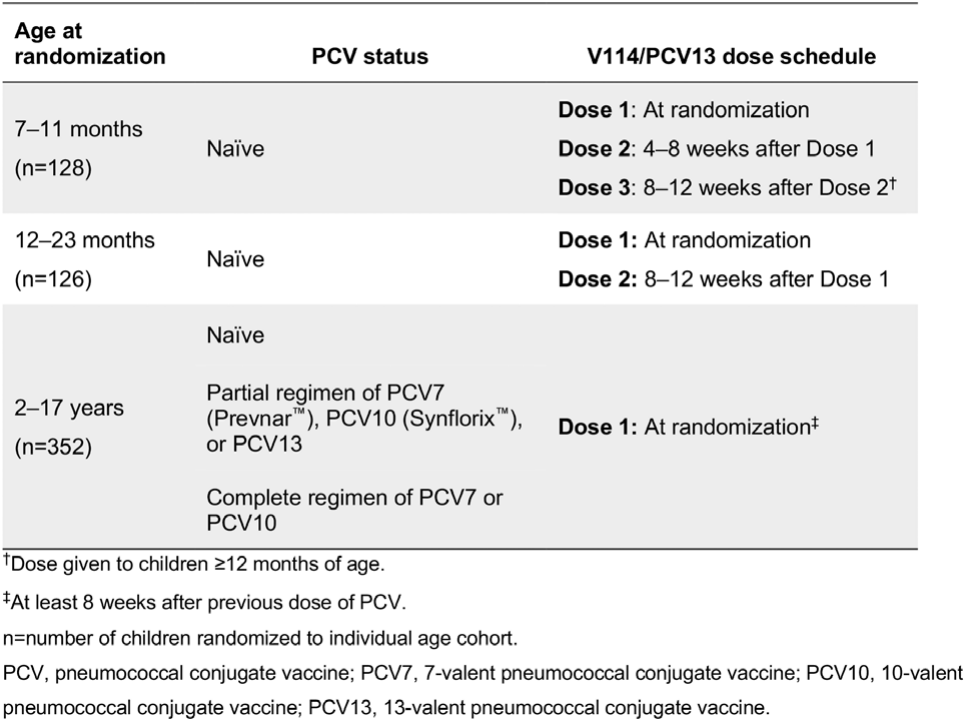

**Conclusion:**

Catch-up vaccination with V114 in healthy children aged 7 months through 17 years had an acceptable safety profile, was well tolerated, and provided comparable immune responses to the 13 serotypes shared with PCV13, and higher immune responses to serotypes 22F and 33F.

**Disclosures:**

**Natalie Banniettis, MD**, **Merck Sharp and Dohme** (Employee, Shareholder) **Jacek Wysocki, MD, PhD**, **GlaxoSmithKline** (Scientific Research Study Investigator, Advisor or Review Panel member, Research Grant or Support)**MSD** (Grant/Research Support, Scientific Research Study Investigator, Advisor or Review Panel member)**Pfizer** (Scientific Research Study Investigator, Advisor or Review Panel member, Research Grant or Support) **Mika Rämet, MD, PhD**, **MSD** (Scientific Research Study Investigator) **Ron Dagan, MD**, **Medimmune/AstraZeneca** (Grant/Research Support, Scientific Research Study Investigator, Research Grant or Support)**MSD** (Consultant, Grant/Research Support, Scientific Research Study Investigator, Advisor or Review Panel member, Research Grant or Support, Speaker’s Bureau)**Pfizer** (Consultant, Grant/Research Support, Scientific Research Study Investigator, Advisor or Review Panel member, Research Grant or Support, Speaker’s Bureau) **Lori Good, B.S.**, **Merck & Co., Inc** (Employee) **Melanie Papa, BA**, **Merck Sharp and Dohme** (Employee, Shareholder) **Yaru Shi, PhD**, **Merck & Co., Inc** (Employee) **Luwy Musey, MD**, **Merck & Co., Inc.** (Employee) **Kara Bickham, MD**, **Merck Sharp and Dohme** (Employee, Shareholder) **Gretchen Tamms, B.S.**, **Merck Sharp and Dohme** (Employee, Shareholder) **Richard McFetridge, B.S.**, **Merck & Co., Inc** (Employee) **Robert Lupinacci, M.S**, **Merck & Co., Inc** (Employee, Shareholder)

